# Finding the region of pseudo-periodic tandem repeats in biological sequences

**DOI:** 10.1186/1748-7188-1-2

**Published:** 2006-02-28

**Authors:** Xiaowen Liu, Lusheng Wang

**Affiliations:** 1 Department of Computer Science, City University of Hong Kong, Kowloon, Hong Kong

## Abstract

**Summary:**

The genomes of many species are dominated by short sequences repeated consecutively. It is estimated that over 10% of the human genome consists of tandemly repeated sequences. Finding repeated regions in long sequences is important in sequence analysis.

We develop a software, LocRepeat, that finds regions of pseudo-periodic repeats in a long sequence. We use the definition of Li et al. [1] for the pseudo-periodic partition of a region and extend the algorithm that can select the repeated region from a given long sequence and give the pseudo-periodic partition of the region.

**Availability:**

LocRepeat is available at

## Background

Finding pseudo-periodic repeats (or tandem repeats) is an important task in biological sequence analysis [1-3]. The genomes of many species are dominated by short sequences repeated consecutively. It is estimated that over 10% of the human genome consists of tandemly repeated sequences. About 10–25% of all known proteins have some form of repeated structure ranging from simple homopolymers to multiple duplications of entire globular domains. An instance (originally from Jaitly et al. [2]) of a human tandem repeat appears below (Genbank:10120313):

CCTCCTCCTCCACCTCCTCCTCCTCCTCCTCCTCCTCCGCCTTCTCATCCTCCTCCACTT

CCTCCTCCTCCTCCTCCTCCCCTTCTCATCCTCCTCCTCTTCATCTACCC

This tandem repeat consists of 35 approximate copies of the repeated pattern CCT.

Variation in the pseudo-periodic repeats demonstrates biologically important information. Sensitive tools for finding those regions containing pseudo-periodic repeats are required in practice. Repeats occur frequently in biological sequences, but they may not be exact in many cases. If the repeats are exact, the problem can be easily solved from computation point of view. However, repeats are seldom exact in biological sequences. The errors in those repeats make it difficult to find regions of those repeats. Many measures and algorithms have been proposed.

Landau and Schmidt [4] studied the problem of finding the two consecutive copies in a sequence of length *n *such that the edit distance (a match costs 0 and a mismatch/indel costs 1) between the two copies is at most *k*. The running time of the algorithm is *O*(*kn *log *k *log(*n*/*kL*)). Schmidt [5] used weighted grid digraphs for finding all non-overlapping pairs of substrings (not necessarily consecutive) with the highest scores in a given string of length *n*. The algorithm can handle any score scheme. It requires *O*(*n*^2 ^log *n*) time and Θ(*n*^2^) space. In both [4] and [5], only two copies of the pattern are considered.

### Measures for finding repeats

Three measures can be used to give partitions of repeated regions.

#### Quasiperiodicty

Wan and Song proposed a measure in which all the repeated copies (except the last one) have the same length [6]. For this measure, a linear time and space algorithm was given [6].

#### Approximate periods

Sim et al. [7] introduced a notion of approximation periods (*approximate period*) using edit distance or relative edit distance. The problem in general is defined as follows: given a string *x*, find a repeated pattern *p *such that *x *can be partitioned as *x *= *p*_1_*p*_2_...*p*_*k *_and  is minimized. Here *d*(*p*, *p*_*l*_) is the relative edit distance which is  the edit distance, where *L *= (|*p*| + |*p*_*l*_|)/2 is the average length of the two strings *p *and *p*_*l*_. Note that, the normalization of the edit distance is important for finding repeated patterns since otherwise, one can give a partition in which each pattern has one letter and the edit distance is at most 1 (small). The problem in general is NP-hard [7]. When the repeated pattern *p *is assumed to be a substring of *x*, The problem can be solved in *O*(|*x*|^4^) time. Note that the second measure is more general than the first since it allows insertions and deletions. Both measures in [7] and [6] use the bottleneck function that finds the repeated pattern *p *and assumes that each copy *p*_*i *_in the long string is close to the repeated pattern *p*, i.e., *d*(*p*_*i*_, *p*) ≤ *δ *and *δ *is minimized. However, in biological sequences, copies of the repeated patterns may change gradually so that some repeats in the region may have very little in common. For example, it is well-known that the N-terminal non-globular region of *Thermus thermophilus *seryl-tRNA synthetase (PDB:1SRY) [1,8] has weak 7-residue repeats. See Table 1. The similarity score between two consecutive patterns is calculated using Blosum62 matrix and the gap penalty is set to be -4. The repeated patterns gradually changes from the 4-th unit LDLEALLA to the 13-th unit KEARLE. The average similarity score for the nine pairs of consecutive patterns is 4.56. But the similarity score between the 4-th unit and the 8-th unit is -11. In this case, the algorithms based on the bottleneck function may fail to find the multiple repeats.

**Table 1:** Pseudo periodic repeats of 1SRY (matrix:blosum62, gap penalty: -4)

Unit	Pseudo-periodic unit	Length	Similarity with previous unit
1	MVDLKRLR	8	
2	QEPEVFHR	8	-5
3	AIREKGVA	8	-10
4	LDLEALLA	8	-1
5	LDREVQEL	8	7
6	KKRLQEVQ	8	6
7	TERNQVA	7	6
8	KRVPKAP	7	-4
9	PEEKEAL	7	-2
10	IARGKAL	7	3
11	GEEAKRL	7	3
12	EEALRE	6	10
13	KEARLE	6	12
14	ALLLQV	6	-6
15	PLPP	4	-8

#### Pseudo-periodic repeats

Li et al. [1] gave the first measure that allows gradual changes of patterns and changes of pattern lengths in the region. The repeats they defined are called the *pseudo-periodic *repeats. Given a repeated region (a string) *x *and a partition *X *= *s*_1_*s*_2_...*s*_*k*_, the *pseudo periodic score *is



where *d*(·) is the edit distance, |*s*_*i*_| is the length of *s*_*i*_, and *c *is a factor that control the penalty of the two ends of the partition. Li et al. [1] gave a *O*(|*x*|^2^) algorithm to compute an optimal partition of a given repeated region *x*. It was shown that the pseudo-periodic score can accurately give partitions for tandem repeated regions, where the repeated patterns are weakly similar.

**Example: **The example is from [1]*. *The sequence of the LbH domain of members of the LpxA family consists of the imperfect tandem repetition of hexapeptide units [9-11]. These imperfect tandem repeats (partitions) have been accurately detected by the algorithm using the pseudo periodic score [1]. (See Table 2).

**Table 2:** Pseudo periodic repeats of LPXA_ECOLI (matrix:blosum62, gap penalty: -4)

Unit	Pseudo-periodic unit	Length	Similarity with previous unit
1	MIDKSAFVHPTAIVEEGA	18	
2	SIGANAHIGPFCIVGPHV	18	14
3	EIGEGTVLKSHVVVNGHT	18	16
4	KIGRDNEIYQFASI	14	-7
5	GEVNQDLKYAGEPTR	15	-3
6	VEIGDRNRIRESVTI	15	2
7	HRGTVQGGGL	10	-14
8	TKVGSDNLLMINAHIAHD	18	-24
9	CTVGNRCILANNATLAGH	18	20
10	VSVDDFAIIGGMTAVHQF	18	4
11	CIIGAHVMVGGCSGV	15	4

In sequence analysis, we may have a long sequence *s *and only a substring *t *(or a few substrings) of *s *contains the consecutive repeats. The problem here is to find out the substring *t *and give an optimal pseudo-periodic partition. We call this problem the *local pseudo-periodic *problem. In this paper, we define the maximization version of the pseudo-periodic partition and develop an algorithm that solves the local pseudo-periodic problem in *O*(*n*^2^) time, where *n *is the length of the input sequence *s*.

## Definitions

In this section, we first give a definition of the *pseudo-periodic partition *of a string that is originally proposed in Li et al. [1]*. *We then give a definition of the *local pseudo-periodic partition *of a string.

### Pseudo-periodic Partition

Let *s *= *a*_1_*a*_2_...*a*_*n *_be a string of length *n*. A *partition π*(*s*) = {*s*_1_, *s*_2_, ..., *s*_*k*_} of s is a set of substrings of *s *such that *s *= *s*_1_*s*_2_...*s*_*k *_(*s*_*i*_'s are also called *repeats*). When *s *is clear, we use *π *instead of *π*(*s*). Π(*s*) denotes the set of all partitions of *s*. Let *s*_*i *_and *s*_*i*+1 _be two strings. The *similarity measure μ*(*s*_*i*_, *s*_*i*+1_) between *s*_*i *_and *s*_*i*+1 _is the maximum alignment value for *s*_*i *_and *s*_*i*+1_. For any two letters (possibly spaces) *x *and *y*, *μ*(*x*, *y*) is the similarity score between the two letters. For example, one can use the following score scheme *I*: a match costs 1, a mismatch costs -1, and an insertion or deletion costs -1. Here we choose to use maximization version since for protein sequences, there are popular similarity matrices, e.g., PAM matrix.

Let *c *be a negative constant. We call  the *granularity factor*. Let Δ denotes a space in an alignment. In this paper, we assume that  >*μ*(*x*, Δ) for any letter *x *in the given sequence.

**Figure 1:**
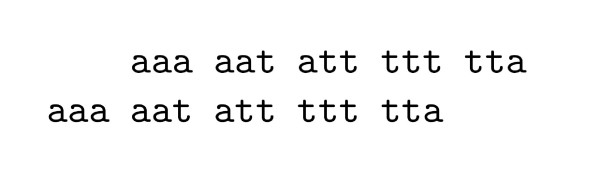
The alignment for *π*(*s*_*A*_).

Let us consider the following example. *s*_*A *_= *s*_1_*s*_2_*s*_3_*s*_4_*s*_5 _and *π*(*s*_*A*_) = {*s*_1_, *s*_2_, *s*_3_, *s*_4_, *s*_5_}, where *s*_1 _= *aaa*, *s*_2 _= *aat*, *s*_3 _= *att*, *s*_4 _= *ttt *and *s*_5 _= *tta*. The self-alignment of this partition is show in Figure 1. The value of the self-alignment is , where |*s*_1_| and |*s*_5_| are the penalty scores for the two segments *s*_1 _and *s*_5 _aligned to spaces.

Note that the score for insertion and deletion would be different from the granularity factor . If there is a gap at the right end of the alignment between *s*_4 _and *s*_5_, there is ambiguity in the calculation of the self-alignment value. Therefore, we need a more precise definition for the value of the self-alignment corresponding to a partition.

Let *s *= *s*_1_*s*_2_...*s*_*k *_be the string and *π*(*s*) = {*s*_1_, *s*_2_, ..., *s*_*k*_}. |*s*| denotes the length of the string. *pre*(*s*, *i*) is the length-*i *prefix of *s *and *suf*(*s*, *i*) is the length-*i *suffix of *s*. Note that the gap at the right end of the self-alignment of *s *only appears in the segments *s*_*k*-1 _and *s*_*k*_. Denote by *s*_*e *_the suffix *s*_*k*-1_*s*_*k *_of *s*. We can designate that only the last *i *letters in *s*_*e *_are mapped to spaces with score  each, for 1 ≤ *i *≤ |*s*_*e*_|. Now let us consider the remain part *pre*(*s*_*e*_, |*s*_*e*_| - *i*) of *s*_*e*_. There are two cases: (1) If *i *≥ |*s*_*k*_|, *pre*(*s*_*e*_, |*s*_*e*_| - *i*) is a prefix of *s*_*k*-1 _and is optimally aligned with *s*_*k*_. (2) If *i *< |*s*_*k*_|, *pre*(*s*_*e*_, |*s*_*e*_| - *i*) contains *s*_*k*-1 _and a prefix of *s*_*k*_. In this case, *s*_*k*-1 _is optimally aligned with *s*_*k *_and the letters in the prefix of *s*_*k *_are scored as *μ*(*x*, Δ) each.

For a partition *π *of *s *and a fixed *i*, 1 ≤ *i *≤ |*s*_*e*_|, let *V*(*π*, *c*, *i*) be the value of the self-alignment such that *s*_1 _is mapped to spaces with score  each, *s*_*j *_is optimally aligned with *s*_*j*+1 _for *j *= 1, 2, ..., *k *-2, *pre*(*s*_*e*_, |*s*_*e*_| - *i*) is scored according to the above two cases, and the last *i *letters in *s*_*e *_are mapped to spaces with score  each. We have



In *V*(*π*, *c*, *i*), the alignment between *s*_1_*s*_2_...*s*_*k*-2_*pre*(*s*_*e*_, |*s*_*e*_| - *i*) and *s*_2_*s*_3_...*s*_*k *_is called the *middle *alignment. The value of the self-alignment of *π *is defined as .

**Figure 2:**
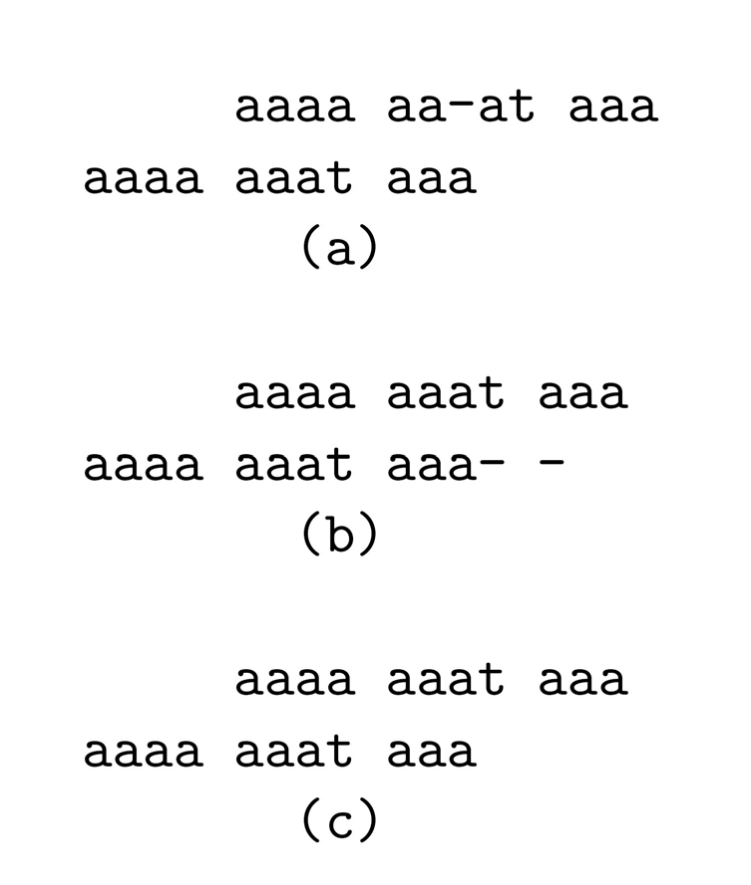
The alignment for *π*(*s*_*B*_).

For example, let *s*_*B *_= *s*_1_*s*_2_*s*_3 _and *π*(*s*_*B*_) = {*s*_1_, *s*_2_, *s*_3_}, where *s*_1 _= *aaaa*, *s*_2 _= *aaat *and *s*_3 _= *aaa*. We use score scheme *I *and *c *= -1. The valid value of *i *is 1, 2, ..., 7 since |*s*_2_*s*_3_| = 7. For *i *= 5 ≥ |*s*_3_|, *pre*(*s*_2_, 2) is optimally aligned with *s*_3_, *s*_1 _and *suf*(*s*_2_*s*_3_, 5) is scored as  (Figure 2(a)). So *V*(*π*(*s*_*B*_), *c*, 5) = *μ*(*s*_1_, *s*_2_) + *μ*(*pre*(*s*_2_, 2), *s*_3_) +  × (|*s*_1_| + 5) = . For *i *= 2 < |*s*_3_|, *s*_2 _is optimally aligned with *s*_3_, *pre*(*s*_3_, 1) is scored as *μ*(*x*, Δ) and *suf*(*s*_3_, 2) is scored as  (Figure 2(b)). In this case, *V*(*π*(*s*_*B*_), *c*, 2) = *μ*(*s*_1_, *s*_2_) + *μ*(*s*_2_, *s*_3_) + *μ*(*x*, Δ) × 1 +  × (|*s*_1_| + 2) = 0. For *i *= 4, at the right ends of the optimal self-alignment of *π*(*s*_*B*_) (Figure 2(c)), there are 4 letters that match spaces. The last letter *t *in *s*_2 _matches a space at the right end of the alignment. The assumption that  >*μ*(*x*, Δ) forces this column to have score  instead of *μ*(*t*, Δ) to maximize *V*(*π*, *c*). We have *V*(*π*(*s*_*B*_), *c*) = *V*(*π*(*s*_*B*_), *c*, 4) = 1. For *i *= 1, 3, 6, 7, the values are lower than *V*(*π*(*s*_*B*_), *c*) = *V*(*π*(*s*_*B*_), *c*, 4) = 1.

Let Π(*s*) be the set of all possible partitions of *s*. *B*_*c*_(*s*) = max_*π*∈Π(*s*) _*V*(*π*, *c*) is the optimal *V*(·) value of partitions. A partition *π*_*q *_= {*s*_1_, *s*_2_, ..., *s*_*k*_} of *s *is called the pseudo-periodic partition of *s *if *B*_*c*_(*s*) = *V*(*π*_*q*_, *c*). In Li et al. [1], it was demonstrated that the numerical measure *B*_*c*_(*s*) (in fact, the minimization version) is sensitive for partitioning *s *into repeats that allow the gradual changes of patterns and changes of pattern lengths.

In practice, we are given a long string *s*. We want to find a region (substring) *t *of *s *that contains pseudo-periodic repeats. Once the region *t *is found, we want to get the pseudo-periodic partition of *t*. The mathematical problem is defined as follows:

### Local pseudo-periodic partition problem

Given a string *s*, find a substring *t *(the local optimal pseudo-periodic region) of *s *such that



where *Sub*(*s*) is the subset of all substrings of *s*.

## The algorithm

Let *s *be the given string. We want to find a substring *t *of *s *with the maximum self-alignment value . Let *s*[1, *j*] be the substring of *s *that consists of the first *j *letters. Informally, we use *w*(*i*, *j*) to denote the maximum self-alignment value of a suffix *t*_*j *_of *s*[1, *j*] such that there are *i *letters at the right end of the self-alignment of *t*_*j *_that are aligned with spaces and scored as . Note that, the right end of the self-alignment of *t*_*j *_could contain more than *i *spaces. However, only the last *i *spaces are scores as  each and the rest of them are scored as the score for *μ*(*x*, Δ).

Let *T*_*j *_be the set of all suffixes of *s*[1, *j*]. For a substring *t *of *s *and an integer *i*, Π(*t*, *i*) = {*π*(*t*)|*π*(*t*) ∈ Π(*t*) and |*t*_*k*-1_*t*_*k*_| ≥ *i*}, where *t*_*k*-1_, *t*_*k *_are the last two repeats in *π*(*t*). We define



to be the maximum *V*(·, *c*, *i*) value of all the partitions in Π(*t*, *i*), where *t *is a substring in *T*_*j*_. To compute *w*(*i*, *j*) using dynamic programming method, we first consider the boundary values of *w*(*i*, *j*). We set *w*(0, *j*) = -∞ since we do not allow *suf*(*t*_*k*-*1*_*t*_*k*_, *i*) to be empty. Note that, by definition, *i *≤ *j*.

**Lemma 1 ***For a sequence s of length n, w*(*j*, *j*) = *c*·*j *for 1 ≤ *j *≤ *n*.

**Proof. **For a partition *π*(*t*) = {*t*_1_, *t*_2_, ..., *t*_*k*_} satisfying *t *∈ *T*_*j *_and *π*(*t*) ∈ Π(*t*, *j*), from the definition of Π(*t*, *j*), |*t*_*k*-1_*t*_*k*_| ≥ *j*. Since *t *is a suffix of *s*[1, *j*] and |*t*| ≥ |*t*_*k*-1_*t*_*k*_| ≥ *j*, we have *t *= *s*[1, *j*] and 1 ≤ *k *≤ 2. Consider the self alignment of *π*(*t*) such that the last *j *letters in *t *are mapped to spaces with score . Two cases arise. Case 1: *k *= 1 and *t *= *t*_1 _= *s*[1, *j*]. In this case, the middle alignment is empty. Thus, *V*(*π*(*t*), *c*, *j*) =  × (|*t*_1_| + *j*) = *c*·*j*. Case 2: *k *= 2 and *t *= *t*_1_*t*_2 _= *s*[1, *j*]. In this case, the middle alignment is the alignment between |*t*_2_| spaces and *t*_2_. By the assumption that  >*μ*(Δ, *x*), *V*(*π*(*t*), *c*, *j*) = |*t*_2_| × *μ*(Δ, *x*) +  × (|*t*_1_| + *j*) <*c*·*j*. □

From the above analysis, the initial and boundary values are

*w*(0, *j*) = -∞, *w*(*j*, *j*) = *c*·*j *    (2)

**Theorem 2 ***For a sequence s of length n and *2 ≤ *j *≤ *n*, 1 ≤ *i *<*j*,



**Proof. **Consider the partition *π*(*t*) = {*t*_1_, *t*_2_, ..., *t*_*k*_} such that *t *∈ *T*_*j*_, *π*(*t*) ∈ Π(*t*, *i*) and *V*(*π*(*t*), *c*, *i*) = *w*(*i*, *j*). We analyze the value of *V*(*π*(*t*), *c*, *i*) based on different cases.

**case 1. ***π*(*t*) has only one repeat. We have |*t*| = |*t*_1_| = *i *and the middle alignment is empty. Therefore *V*(*π*(*t*), *c*, *i*) =  × (|*t*_1_| + *i*) = *c*·*i*.

**case 2. ***π*(*t*) has *k *≥ 2 repeats. In this case, the middle alignment is not empty since it contains *t*_*k*_. The last column in the middle alignment has three configurations: (a) the last column contains two letters *s*[*j *- *i*] and *s*[*j*], (b) the last column contains a space and the letter *s*[*j*], (c) the last column contains the letter *s*[*j *- *i*] and a space. For sub-case (a), if we take away the last letter *s*[*j*] from the self alignment of *π*(*t*), we can get a self alignment of *π*'(*t*'), where *t*' ∈ *T*_*j*-1_, *π*'(*t*') ∈ Π(*t*', *i*) and *V*(*π*'(*t*'), *c*, *i*) = *w*(*i*, *j *- 1). By comparing the two self alignment, we have *V*(*π*(*t *, *c*, *i *= *V*(*π*'(*t*'), *c*, *i*) + *μ*(*s*[*j *- *i*], *s*[*j*]) = *w*(*i*, *j *- 1) + *μ*(*s*[*j *- *i*], *s*[*j*]). For sub-case (b), if we take away the last letter *s*[*j*] and space aligned with *s*[*j*] from the self alignment of *π*(*t*), we can get a self alignment of *π*'(*t*'), where *t*' ∈ *T*_*j*-1_, *π*'(*t*') ∈ Π(*t*', *i *- 1) and *V*(*π*'(*t*'), *c*, *i*) = *w*(*i *- 1, *j *- 1). Notice that in the end of the self alignment of *π*'(*t*'), there are only *i *- 1 letters mapped to spaces with score , and there is one more letter in the self alignment of *π*(*t*) mapped to spaces with score . Thus, *V*(*π*(*t*), *c*, *i*) = *w*(*i *- 1, *j *- 1) + *μ*(Δ, *s*[*j*]) + . For sub-case (c), *π*(*t*) ∈ Π(*t*, *i *+ 1). We can impose that the alignment of the letter *s*[*j *- *i*] and the space is scored as , not *μ*(*s*[*j *- *i*], Δ). From *V*(*π*(*t*), *c*, *i *+ 1) = *w*(*i *+ 1, *j*), we have *V*(*π*(*t*), *c*, *i*) = *w*(*i *+ 1, *j*) + *μ*(*s*[*j *- *i*], Δ) - . □

Based on Theorem 2, a dynamic programming algorithm is designed. Let *n *be the length of the input sequence *s*. We compute *w*(*i*, *j*) in the order shown below:

**for ***j *= 1 **to ***n ***do**

**for ***i *= j **downto **1 **do**

compute *w*(*i*, *j*) based on Theorem 2.

Obviously, the time complexity is *O*(*n*^2^), where *n *is the length of the whole string. A standard backtracking process allows us to find the local optimal pseudo-periodic region *t*.

**Figure 3:**
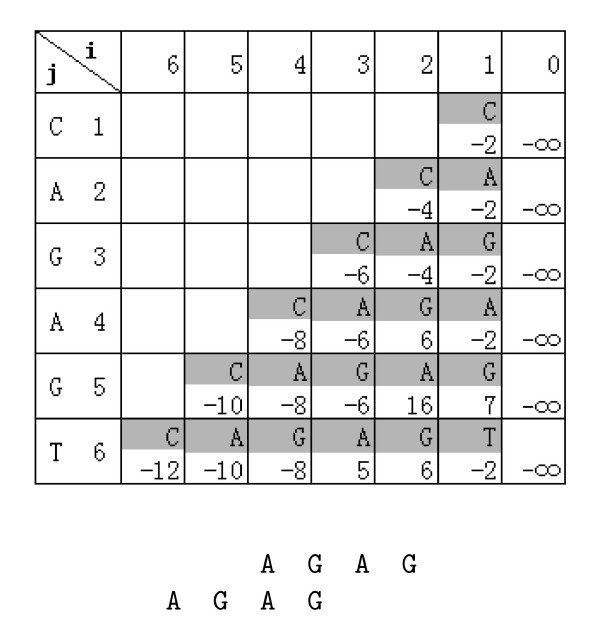
Dynamic programming algorithm and local alignment for s = CAGAGT.

The following example illustrates the algorithm. Let *s *= *CAGAGT*. We set *c *= -2 and use the following score scheme: a match costs 10, a mismatch costs -10, and an insertion or a deletion costs -10. The table constructed by using the dynamic programming algorithm is shown in Figure 3. The table is constructed in from the top to the bottom. For every row in the table, the *w*(*i*, *j*)'s are computed from left to right. From the table, it is easy to see that the maximum value of *w*(*i*, *j*) is *w*(2, 5) = 16. From the maximum value *w*(2, 5) = 16, we know that the local optimal pseudo-periodic region *t *is a suffix of *s*[1, 5] = *CAGAG *and there are 2 letters aligned with spaces and scored as  at the right end of the self alignment of the local optimal pseudo-periodic region *t*. From *w*(2, 5), we can backtrack *w*(2, 5) → *w*(2, 4) → *w*(2, 3) and stop at *w*(2, 3) since *w*(2,3) gets its value from *c*·*i *indicating the first segment of the partition of *t *ends at 3-th letter in *s *and the length of the segment is 2. Thus, we get *t *= *AGAG*. From the self alignment, it is easy to get the partition of *π*(*t*) = {*AG*, *AG*}.

The space complexity required is also *O*(*n*^2^) if we are not careful. However, we can release the space whenever they are no longer useful. Thus, only two columns, are required for the computation. For each of the two columns, we use two arrays: one array stores the value of *w*(*i*, *j*) and the other array stores the starting position of the subsequence *t *that maximizes *w*(*i*, *j*). Therefore, the space complexity is *O*(*n*) for computing all *w*(*i*, *j*)'s. After *w*(*i*, *j*)'s are computed, we know the substring *t *that leads to the optimal *w *value. Therefore, we can reconstruct the alignment for *t *in  time and space, where *n*_1 _is the length of *t*, the repeated region. If *n*_1 _is still big, we can use the standard technique in [12] to reduce the space to *O*(*n*_1_) by doubling the computation time for reconstructing the alignment of *t*.

In practice, a sequence may contain more than one repeated region. To find all the repeated regions, we can select the best *k *values of *w*(*i*, *j*)'s for some pre-defined value *k*. Each backtracking gives a repeated region. Another way to set a threshold for the value of *w*(*i*, *j*) and select all *w*(*i*, *j*)'s with value greater than the threshold.

## Implementation

**Figure 4:**
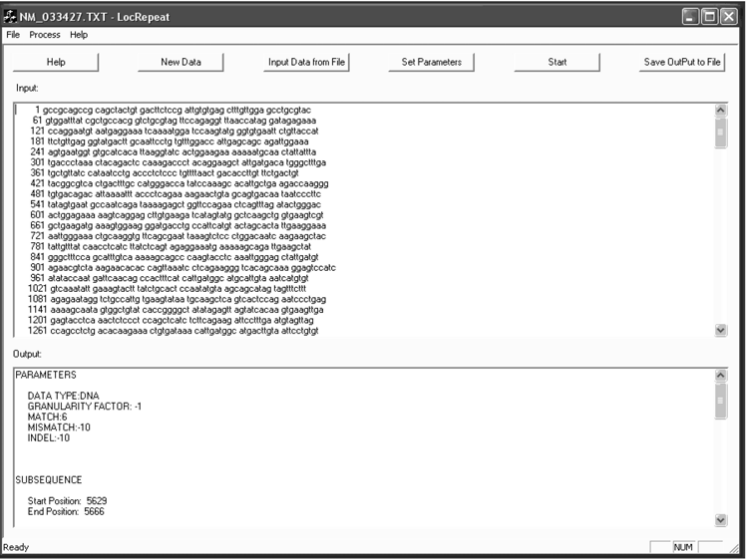
LocRepeat interface.

We have implemented the algorithm using Visual C++ 6.0 and Windows XP. The software is called LocRepeat and has a user-friendly GUI (See Figure 4). Another version without GUI that works for Linux is also available.

LocRepeat accepts three kinds of sequence: DNA, RNA and Protein. The user can either click 'New Data' button to directly input the sequence at the input area, or click 'Input Data from File' button to input a sequence from a file. The user can click 'Set Parameters' button to set parameters, such as granularity factor, gap penalty and similarity score matrix. After the sequence is input and the parameters are set, click the 'Start' button to begin the computation.

## Experiment results

We have done experiments to test the speed and sensitivity of the software.

### Speed Testing

The time complexity of the algorithm is *O*(*n*^2^). To test the speed in practice, we use arbitrarily generated DNA and protein sequences. We ran our software on a PC with Pentium 4 3.4G CPU and 1GB memory, the result is shown in Table 3. We can see that for long DNA and protein sequences, our software can get the result in short time. For example, if the length of the sequence is 10000, it takes about 10.8 seconds and 26.9 seconds for DNA sequences and protein sequences, respectively. In some real applications, the length of sequences could be much longer than 10000. In this case, one can cut the long sequence into several short pieces and find out the repeated regions for each piece. If a region covers two pieces, then we can re-cut that segment to get that region.

**Table 3:** Results for the speed test of LocRepeat

Length	2000	4000	6000	8000	10000
DNA	0.5s	2.2s	4.8s	7.0s	10.8s
PROTEIN	1.1s	4.3s	10.0s	17.7s	26.9s

### Sensitivity testing using real data

We applied LocRepeat to the DNA sequence gene PRNP which contains tandem repeats (GenBank:M13667). The length of the sequence is 2420. We find the local optimal pseudo-periodic region [215,327], that contains 5 pseudo-periodic units (Table 4). The pseudo-periodic region misses the first several sites of the tandem repeats, but the region and the partitions show the tandem repeats correctly.

We also applied LocRepeat to the protein sequence LGR6 (Swiss-Prot: Q9HBX8). The length of the sequence is 828. We use PAM120 as the similarity score matrix and find the local units (Table 5).

**Table 4:** Local optimal pseudo-periodic region for PRNP

Unit	Position	Length	Unit
1	215–238	24	ggtggtggctgggggcagcctcat
2	239–262	24	ggtggtggctgggggcagcctcat
3	263–286	24	ggtggtggctgggggcagccccat
4	287–310	24	ggtggtggctggggacagcctcat
5	311–327	17	ggtggtggctggggtca

**Table 5:** Local optimal pseudo-periodic region for LGR6

Unit	Position	Length	Unit
1	30–53	24	LSMNNLTELQPGLFHHLRFLEELR
2	54–77	24	LSGNHLSHIPGQAFSGLYSLKILM
3	78–101	24	LQNNQLGGIPAEALWELPSLQSLD
4	102–124	23	LNYNKLQEFPVAIRTLGRLQELG
5	125–148	24	FHNNNIKAIPEKAFMGNPLLQTIH
6	149–172	24	FYDNPIQFVGRSAFQYLPKLHTLS
7	173–195	23	LNGAMDIQEFPDLKGTTSLEILT
8	196–219	24	LTRAGIRLLPSGMCQQLPRLRVLE
9	220–241	22	LSHNQIEELPSLHRCQKLEEIG
10	242–265	24	LQHNRIWEIGADTFSQLSSLQALD
11	266–289	24	LSWNAIRSIHPEAFSTLHSLVKLD
12	290–311	22	LTDNQLTTLPLAGLGGLMHLKL

In conclusion, the algorithm presented in this paper offers the possibility to find regions of pseudo-periodic repeats in a long sequence.
